# Options for modulating intra-specific competition in colonial pinnipeds: the case of harbour seals (*Phoca vitulina*) in the Wadden Sea

**DOI:** 10.7717/peerj.957

**Published:** 2015-06-09

**Authors:** Rory P. Wilson, Nikolai Liebsch, Agustina Gómez-Laich, William P. Kay, Andrew Bone, Victoria J. Hobson, Ursula Siebert

**Affiliations:** 1 Swansea Laboratory for Animal Movement, Biosciences, College of Science, Swansea University, Swansea, Wales, UK; 2 GEOMAR Helmholtz Centre for Ocean Research, Düsternbrooker, Kiel, Germany; 3 Customized Animal Tracking Solutions, Moffat Beach, QLD, Australia; 4 Centro Nacional Patagonico-CONICET, Puerto Madryn (U9120ACD), Chubut, Argentina; 5 Institute for Terrestrial and Aquatic Wildlife Research, University of Veterinary Medicine Hannover, Büsum, Germany

**Keywords:** Pinniped, Dive behaviour, Sexual segregation, Dive duration, Swim speed, Harbour seal

## Abstract

Colonial pinnipeds may be subject to substantial consumptive competition because they are large, slow-moving central place foragers. We examined possible mechanisms for reducing this competition by examining the diving behaviour of harbour seals (*Phoca vitulina*) after equipping 34 seals (11 females, 23 males) foraging from three locations; Rømø, Denmark and Lorenzenplate and Helgoland, Germany, in the Wadden Sea area with time-depth recorders. Analysis of 319,021 dives revealed little between-colony variation but appreciable inter-sex differences, with males diving deeper than females, but for shorter periods. Males also had higher vertical descent rates. This result suggests that males may have higher overall swim speeds, which would increase higher oxygen consumption, and may explain the shorter dive durations compared to females. Intersex variation in swim speed alone is predicted to lead to fundamental differences in the time use of three-dimensional space, which may help reduce consumptive competition in harbour seals and other colonial pinnipeds.

## Introduction

Competition is a major driver explaining patterns of space use in animals (MacArthur, 1958; Pianka, 2011) and it is considered particularly severe in central-place foraging (*sensu* Orians & Pearson, 1979) and colonial breeders, such as seabirds or pinnipeds, because the density of animals around the breeding site leads to correspondingly high local pressure on resources (e.g., see Birt et al., 1987; Lewis et al., 2006; Gaston, Ydenberg & Smith, 2007; Breed, Bowen & Leonard, 2013). There are a number of mechanisms proposed to reduce consumptive pressure in central-place foraging marine vertebrates. These relate primarily to different individuals taking either different types of prey (Forero et al., 2002; Ishikawa & Watanuki, 2002; Garthe et al., 2007) or the same prey in different spaces (e.g., Wilson, 2010). Both of these are fostered by differences in area use (Thompson et al., 1998), depths (Ishikawa & Watanuki, 2002; Laich et al., 2012) and even the timing of foraging bouts (Harris et al., 2013). It has been noted, though, that spatial and temporal segregation in resource use does not necessarily guarantee a reduction in competition (Wilson, 2010).

Competition should be most apparent intra-specifically because individuals of many species are essentially morphologically and behaviourally identical. In addition, while inter-specific competition can theoretically lead to species extinction (Bengtsson, 1989; Andersen et al., 2007), intra-specific (and particularly inter-gender) competition cannot. It is partly for this reason, therefore, that there is presumably strong selection pressure for central-place foraging, colonial species to exhibit sexual differences in foraging behaviour. In many marine, air-breathing, diving vertebrates these differences in foraging behaviour can be driven by size, because allometric effects modulate mass-specific metabolic rate (Kleiber, 1932; Schmidt-Nielsen, 1997), body oxygen stores (Halsey, Butler & Blackburn, 2006; Brischoux et al., 2008), fasting capacity (Lindstedt & Boyce, 1985) and a suite of other performance-related parameters (see Peters, 1983). Unsurprisingly, it is specifically the gender-related differences in body mass that have been linked to sex-dependent differences in depth use in both seabirds, such as Imperial cormorants (*Phalacrocorax atriceps*; Laich et al., 2012) and Galapagos cormorants (*Phalacrocorax harrisi*; Wilson et al., 2008), and pinnipeds, such as Northern elephant seals (*Mirounga angustirostris*: Le Boeuf et al., 2000) and New Zealand fur seals (*Arctocephalus forsteri*; Page, McKenzie & Goldsworthy, 2005). Indeed, sex-dependent difference in foraging is considered to be a major contribution to minimizing the effects of competition, not least because bottom-foraging species distributing themselves according to depth, also do so with regard to foraging area (e.g., Quintana et al., 2011). However, not all colonial, central-place foraging diving vertebrates are sexually dimorphic, which should mean, in terms of foraging at least, that there are no differential capacities with regard to depth use, for example. How then, might niche partitioning be achieved?

We studied the foraging behaviour of the harbour seal (*Phoca vitulina*). This species is a ubiquitous, slightly dimorphic species, with the male body mass being some 20% greater than the female (Riedman, 1990; Lindenfors, Tullberg & Biuw, 2002), which forms large stable colonies in the Northern Hemisphere (Ross et al., 2004). Specifically, we used animal-borne multiple channel loggers to determine if there were any obvious gender-dependent differences. Given the very modest dimorphism between sexes compared to many pinnipeds (Lindenfors, Tullberg & Biuw, 2002), we hypothesized that there would be no inter-sex difference in depth use but expected that there may be some evidence of differential foraging behaviour.

## Materials and Methods

### Location and devices

**Figure 1:**
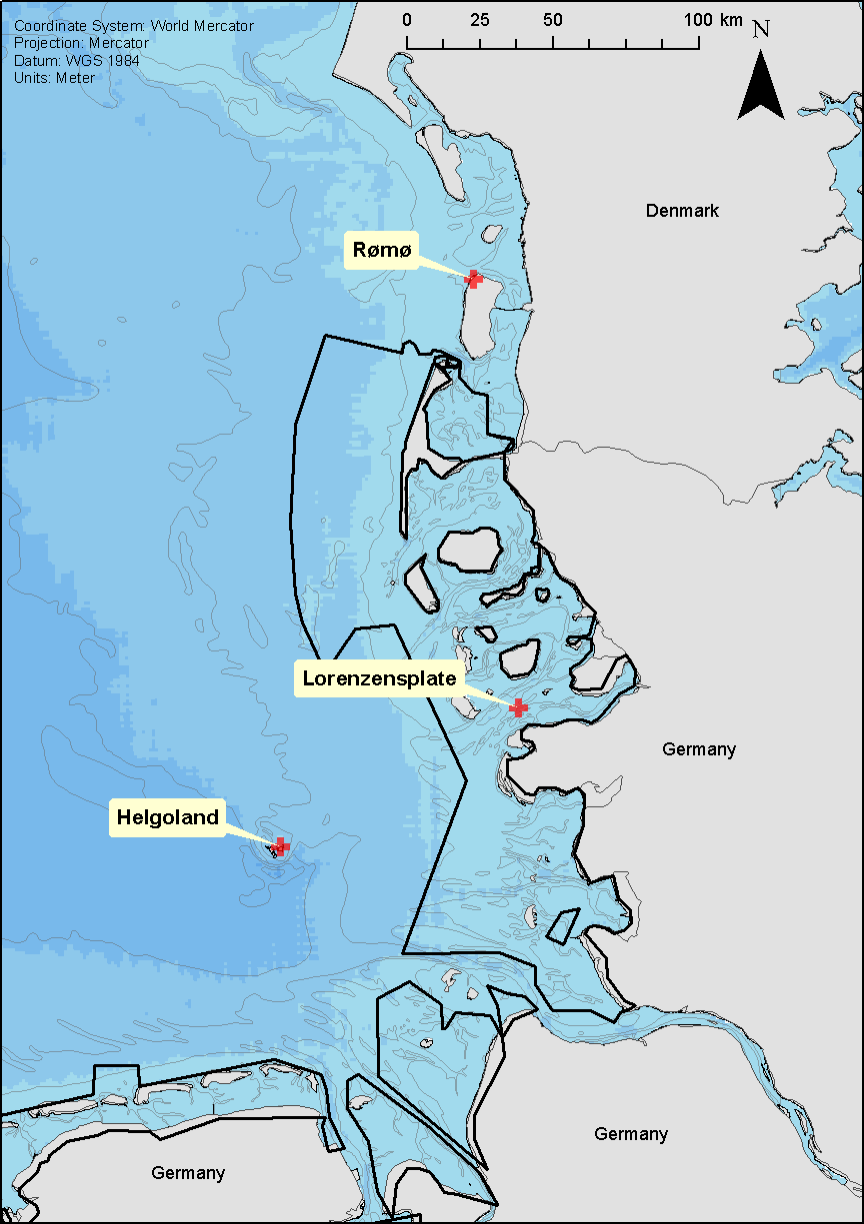
Location of seal captures. Location of the three seal capture locations; Rømø (DK), Lorenzenplate (LP) and Helgoland (HE). The shading of the sea area corresponds to 10 m depth intervals and the continuous black lines show the boarder of the Wadden Sea National Parks of Schleswig-Holstein and Lower Saxony.

Between June 2004 and December 2006, 73 seals were equipped with tags at three well-established haul-out sites within the Wadden Sea and adjacent offshore areas; the Lorenzenplate (54.38°N, 8.53°E—hereafter ‘LP’), Rømø (55.21°N, 8.50°E—hereafter ‘DK’) and Helgoland (54.19°N, 7.92°E—hereafter ‘HE’) (Fig. 1). The tags (140 g, 90 × 65 × 28 mm) were multiple channel loggers (Driesen and Kern GmbH, Bad Bramsted, Germany) recording dive depth, heading, body orientation, temperature and light, and capable of storing up to 32 megabytes of data in a flash RAM with 16 bit resolution at sampling intervals between 1 s and 24 h (Liebsch, 2006). We used a sampling interval of 5 s which nominally allowed a recording period of up to 94 days. The loggers were deployed in tandem with a quartz crystal-controlled timers (60 g, 75 × 37 × 20 mm), able to release the tags from the backs of the animals by burning through a nylon filament that held the main package, which consisted of the timer and the logger within a specially moulded, hydro-dynamically shaped flotation package (280 g, 210 × 90 × 65 mm) made of a 70:30 mix of resin and hollow beads (for details see Liebsch, 2006), from a neoprene pocket stuck to the seal’s back.

### Seal capture

Helgoland seals, which are well-accustomed to tourists, were approached and caught by hand, while animals on Lorenzenplate and Rømø were captured using seine netting techniques (Jeffries, Brown & Harvey, 1993). The tagging did not cover any periods of lactation or breeding. Many more seals were captured than were tagged, and all animals, whether tagged or not, were given a veterinary examination to ensure that only healthy animals judged to be adults were tagged (permit number for animal experiments V312-72241.121-19 (70-6/07); Ministry of Energy, Agriculture, the Environment and Rural Areas Schleswig-Holstein, Germany). Target seals were then strapped to a specifically designed bench to assist with device attachment and to reduce handling stress. First, the device attachment site, which was on the posterior two thirds of the dorsal midline (cf. Bannasch, Wilson & Culik, 1994), was cleaned with seawater to remove excess sand, and then cleaned and dried with acetone. The legs of the neoprene pocket containing the tags (see above) were then glued to the animal using Devcon epoxy (Danvers, Massachusetts, USA). After release, the packages were either released from the seal by the timer or were shed during the moult. Following device separation, most drifted ashore, intended for pick up by beach walkers, who would return the package to the specified address.

### Statistical analysis

All submersions deeper than 1 m were considered as dives. Dives were divided into two categories; ‘U-dives’ or foraging dives, in which the bottom phase lasted more than 5 s (Schreer & Testa, 1996), and all other dives, which included ‘V-dives’ and ‘parabolic’ dives (see Le Boeuf et al., 1992; Sala et al., 2011 for definitions). However, since more than 94% of the dives were classified as U-dives, statistical analyses were only performed on these submersions. For each U-dive, the following parameters were calculated: maximum depth, total duration, bottom duration and the vertical velocity of each dive phase (i.e., descent, bottom and ascent). Differences in these parameters between sites and sexes were tested using linear mixed effects models (LMM) fitted by restricted maximum likelihood (REML). In these models, diving characteristics were set as response variables and individual as a random effect. All models were run using the R package nlme (Pinheiro et al., 2011). Differences in the vertical velocity between diving phases were also tested using a mixed effect model. In this case, the vertical velocity was set as a response variable, the dive phase as the predictor and the individual as a random factor. Mixed effect models were also used to test the effect of maximum dive depth, sex and site on dive duration. Finally, the effect of body parameters (i.e., weight and length) on the diving behaviour was analyzed using linear models whereby sex and site were also included as explanatory variables. All statistical analyses were performed using the open source statistical package R version 2.13.0 (R Development Core Team, 2011). For those parameters with several measurements from individuals, mean values were obtained by calculating a mean over the means from each individual. These values are shown with their standard deviation.

## Results

Of the 73 devices deployed, 55 were recovered (75%) of which 34 registered at least one complete foraging trip (the only results presented herein; Table 1) comprising a total of 301,778 U-dives (Data S1). Males appeared to return more often without a full data set than females (Table 1), for reasons that are unclear.

**Table 1:** Number of devices deployed and recovered from harbour seals with usable data (containing data from at least one full foraging trip) from the three study sites.

Location	Sex	No. equipped	No. recovered	No. with data
Lorenzenplatte	Male	18	14	8
	Female	12	9	7
Rømø	Male	24	15	7
	Female	1	1	1
Helgoland	Male	15	13	8
	Female	3	3	3
**TOTAL**		**73**	**55**	**34**

### Sexual segregation of foraging behaviour

#### Depth utilisation

**Figure 2:**
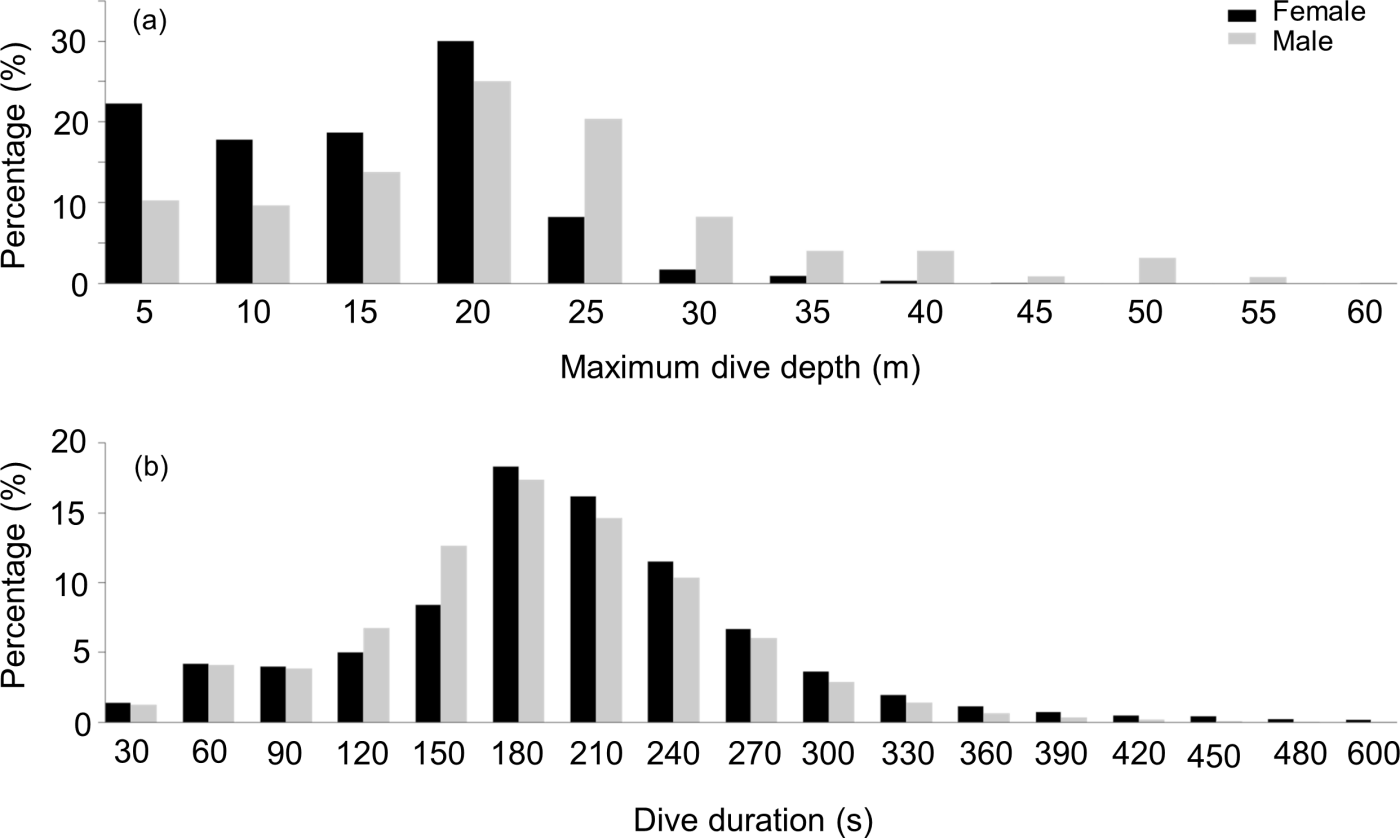
Frequency distribution of the maximum depth and dive duration of U-dives. Frequency distribution of (A) the maximum depth (B) and dive duration of U-dives performed by female (black bars) and male. (grey bars) harbour seals

During their U-dives, males went significantly deeper than females (LMM, Sex effect *F*_1,31_ = 4.4, *p* = 0.04) (Fig. 2A), with no differences in these depths between the three studied sites (LMM, Site effect *F*_1,31_ = 0.01, *p* = 0.9). Mean maximum dive depths were 17.5 ± 2.5, 13.4 ± 3.6 and 19 ± 9 m for males, compared with 12.6, 13.4 ± 3.4 and 8.8 ± 3.5 m for females, from DK, LP and HE, respectively.

#### Dive durations

**Figure 3:**
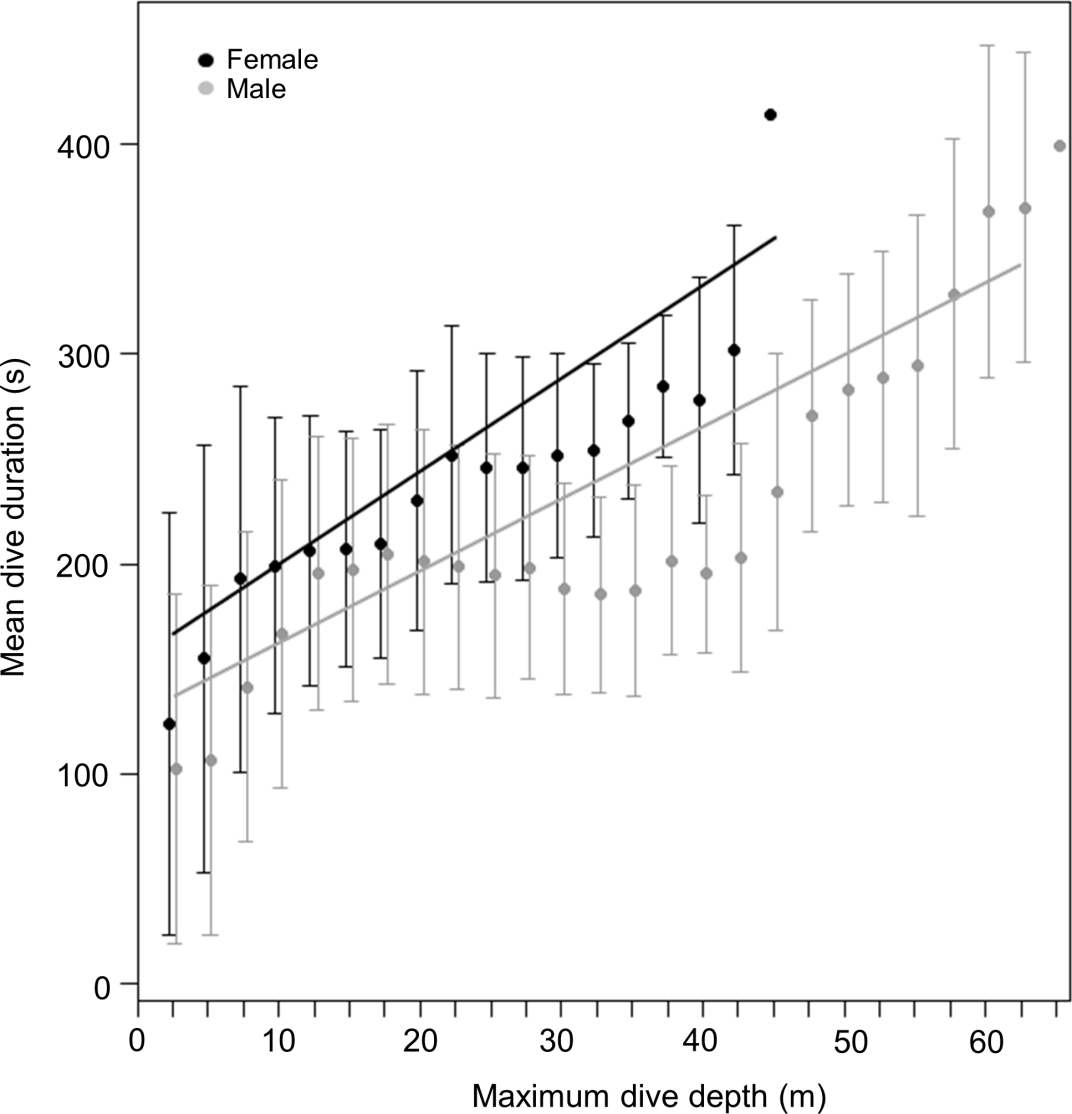
Relationship between dive duration and maximum dive depth of female (black points) and male (grey points) harbour seals. The lines (black for females and grey for males) represent the fitted relationship. The points represent the mean dive duration for 2.5 m interval while the whiskers represent the standard deviation.

During dives, both sexes spent a similar amount of time underwater (LMM, Sex effect *F*_1,31_ = 2.4, *p* = 0.1) (Fig. 2B) with no differences observed between the three locations (LMM, Site effect *F*_1,31_ = 0.07, *p* = 0.8). Mean maximum dive durations were 174 ± 34.6, 199 ± 31 and 185 ± 51 s for males and 213, 211 ± 36.3 and 198 ± 9.8 s for females, from DK, LP and HE, respectively. Deeper dives were accompanied by longer durations, although the relationship between these parameters differed between sexes (Fig. 3). During shallow dives, males and females spent a similar amount of time underwater, however, as dives became deeper, females tended to remain longer underwater than males (LMM, Depth effect *F*_1,301742_ = 65,328, *p* < 0.001; Sex effect *F*_1,32_ = 9, *p* = 0.006; Sex*Depth effect *F*_1,301742_ = 1,018, *p* < 0.001) (*y* = 3.3*x* + 131 and *y* = 4.5*x* + 153 for males and females, respectively).

#### Vertical velocities

There was a significant difference in the rate of change of depth between dive phases as a whole (LMM, Phase effect *F*_1,905298_ = 1,664,062, *p* = 0.006), with the general mean value for the bottom phase being 0.005 ± 0.003 m/s compared with 0.5 ± 0.2 and 0.6 ± 0.2 m/s for the descent and ascent rates, respectively. Males descended and ascended the water column at higher rates than females (Sex effect *F*_1,31_ = 8.0, *p* = 0.008; Sex effect *F*_1,31_ = 8.4, *p* = 0.006; for the descent and ascent phase, respectively) and the same pattern was observed in the three studied sites (Site effect *F*_1,31_ = 0.7, *p* = 0.4; Site effect *F*_1,31_ = 2.9, *p* = 0.09; for the descent and ascent phase, respectively). Mean male descent rates were 0.7 ± 0.1, 0.6 ± 0.1 and 0.7 ± 0.2 m/s compared with 0.5, 0.6 ± 0.1 and 0.4 ± 0.1 m/s for the females, for DK, LP and HE, respectively. The rate of change of depth during the bottom phase was similar between sites (Site effect *F*_1,31_ = 0.1, *p* = 0.7) with values of 0.007 ± 0.002, 0.003 ± 0.003 and 0.007 ± 0.003 m/s for the males and 0.004, 0.003 ± 0.003 and 0.003 ± 0.001 for the females, for DK, LP and HE, respectively.

#### Body weight and diving behaviour

**Figure 4:**
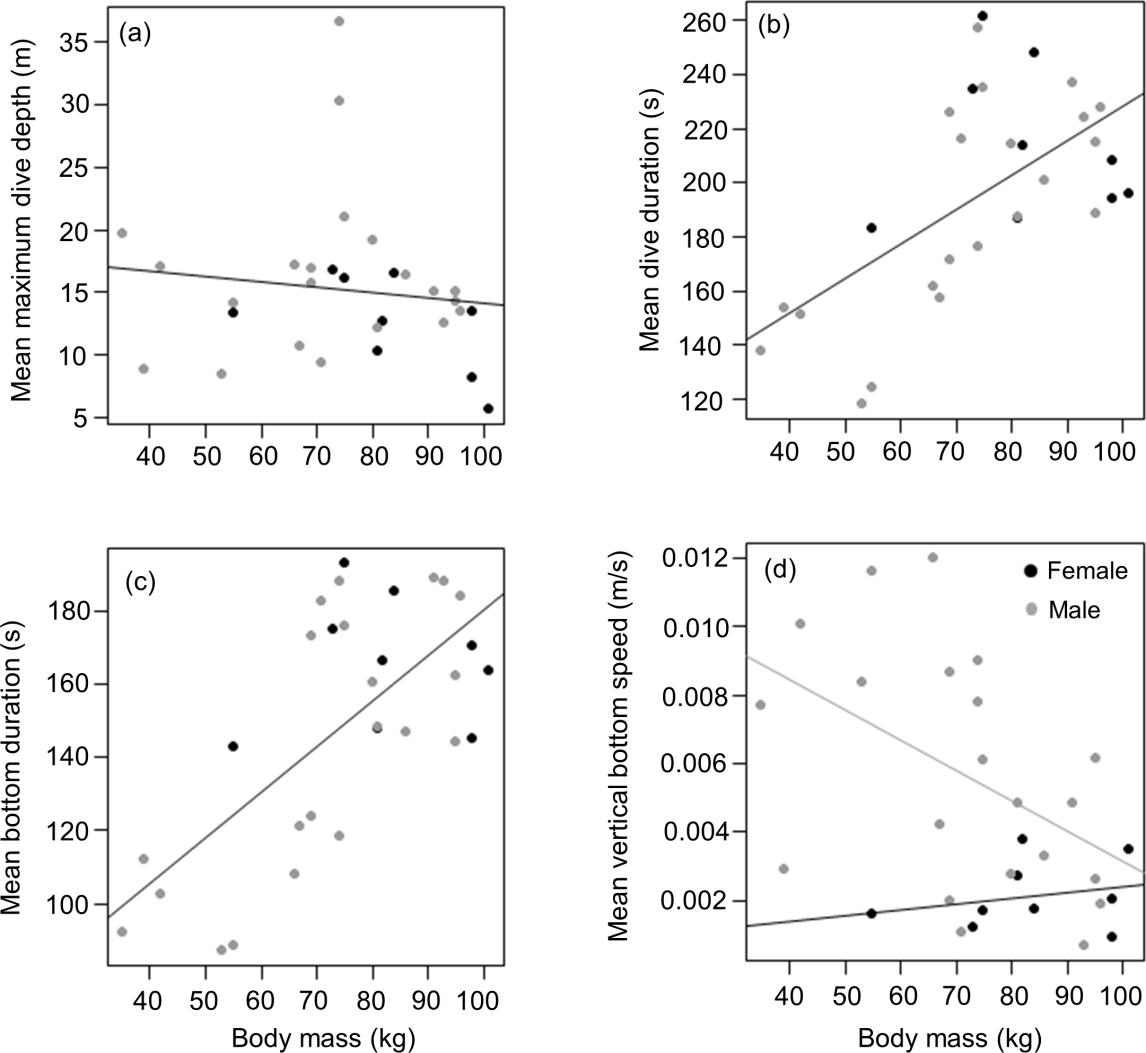
Relationship between (A) mean maximum dive depth (B) mean dive duration (C) mean bottom duration and (D) mean bottom speed as a function of body mass of female and male harbour seals. For those cases where the relationship between sexes differed, female and male points and the fitted line are shown in black and grey, respectively.

Mean masses of tagged males and females from the three different colonies were (males) 69.4 ± 22 (*n* = 5), 74.6 ± 22.8 (*n* = 8) and 70.2 ± 13.1 (*n* = 8) kg *vs* (females) 82.0 (*n* = 1), 77.7 ± 14.2 (*n* = 6) and 99.5 ± 2.1 (*n* = 2) kg for animals from DK, LP and HE, respectively. Overall, there was no significant difference between male and female body weight in our tagged animals (*t* = 1.8, df = 19, *p* = 0.091), males having a mean weight of 71.7 ± 18.5 kg compared to 83 ± 14.7 kg for females. This result was in stark contrast to patterns shown by all seals captured (most were not tagged) during the study. Here, of 377 animals caught, males were significantly heavier than females (means of 70.5 ± 17.7 kg *vs* 62.0 ± 15.7 kg, respectively; Mann–Whitney *W* = 19,461, *p* < 0.01) although there was no specific separation into adult and sub-adult animals undertaken (Data S2). Of the tagged animals, neither females nor males showed a significant relationship between body weight and mean maximum dive depth (*r*^2^ = 0.02, *p* = 0.5; Weight effect *F*_1,29_ = 0.4, *p* = 0.5; Sex effect *F*_1,28_ = 2.1, *p* = 0.2) (Fig. 4A). However, body weight was strongly and positively related with mean dive duration with the relationship between both parameters, being similar for both sexes (*y* = 1.3*x* + 100.7; *r*^2^ = 0.4, *p* < 0.01; Weight effect *F*_1,29_ = 16.2, *p* < 0.001; Sex effect *F*_1,28_ = 0.7, *p* = 0.4) (Fig. 4B). For both sexes, a similar positive relationship was also observed between the mean bottom duration and body weight (*y* = 1.3*x* + 55.9; *r*^2^ = 0.5, *p* < 0.001; Weight effect *F*_1,29_ = 23.9, *p* < 0.001; Sex effect *F*_1,28_ = 0.9, *p* = 0.4) (Fig. 4C). Neither the descent or the ascent rate (change in depth) was affected by body weight (Weight effect *F*_1,29_ = 0.3, *p* = 0.6; Sex effect *F*_1,28_ = 4.2, *p* = 0.05; Weight effect *F*_1,29_ = 0.2, *p* = 0.6; Sex effect *F*_1,28_ = 4.3, *p* = 0.05; for the descent and ascent phases respectively). The rate of change of depth during the bottom phase decreased with increasing body weight but this relationship was only significant for males (*y* = − 8.9.10^−5^*x* + 1.2.10^−2^, *r*^2^ = 0.3, *p* = 0.03; Weight effect *F*_1,28_ = 5.1, *p* = 0.03; Sex effect *F*_1,29_ = 8.8, *p* = 0.006) (Fig. 4D)

#### Body length

**Figure 5:**
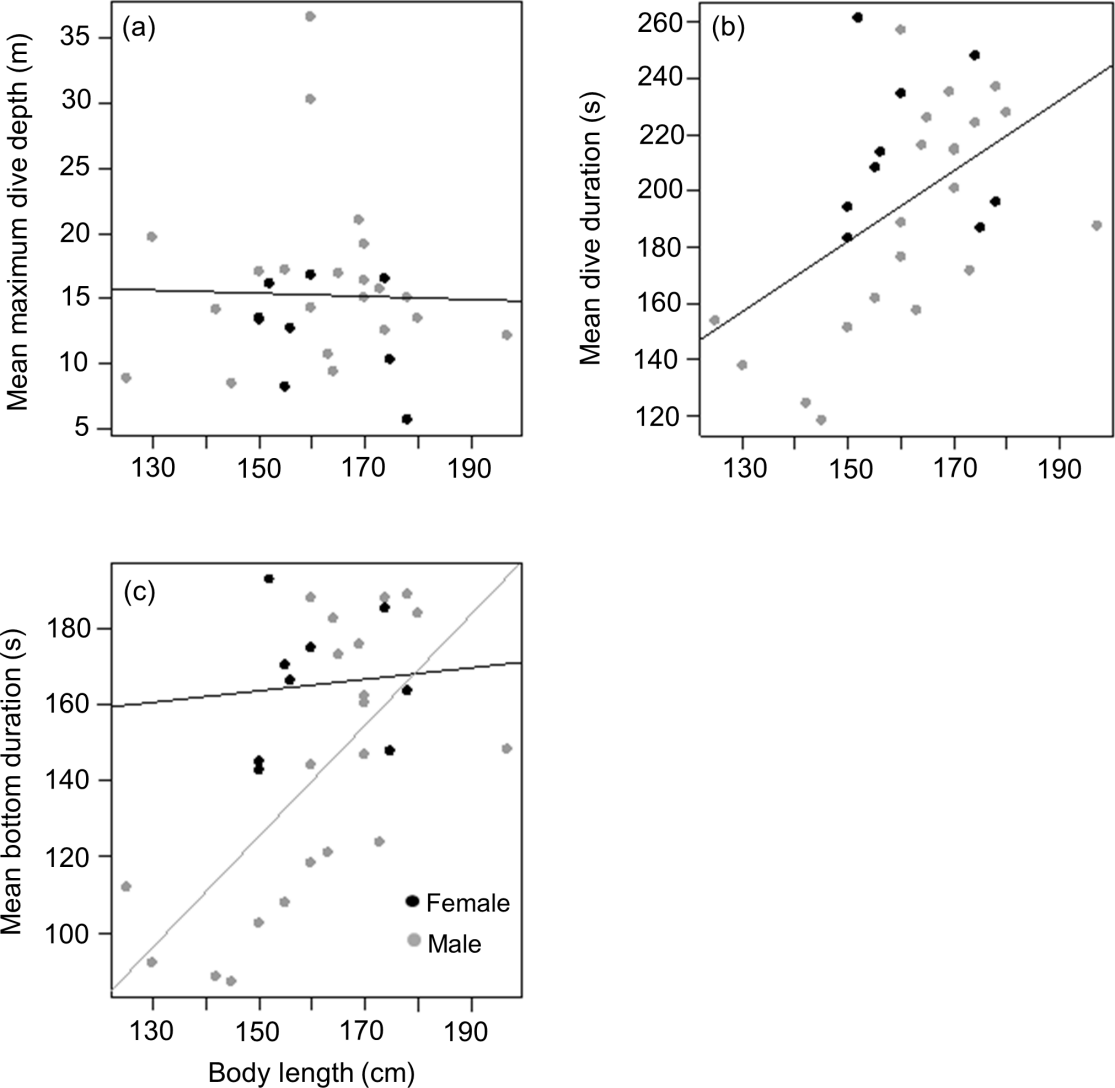
Relationship between (A) mean maximum dive depth (B) mean dive duration and (C) mean bottom duration as a function of body length of female and male harbour seals. For those cases where the relationship between sexes differed, female and male points and the fitted line are shown in black and grey, respectively.

Mean length of tagged males and females from the three different colonies were (males) 161.2 ± 19.4 (*n* = 5), 166 ± 21.4 (*n* = 8) and 158 ± 9.5 (*n* = 8) kg *vs* (females) 156 (*n* = 1), 160 ± 11.7 (*n* = 6) and 166 ± 16.3 (*n* = 2) kg for animals from DK, LP and HE, respectively. As with weight, there was no significant difference between male and female body length in the tagged animals (*t* = − 0.2, df = 22, *p* = 0.9), males having a mean body length of 1.62 ± 0.17 m compared to 1.61 ± 0.11 m for females. Again, this contrasted with the full capture data (377 animals) where males were significantly longer than females (1.62 ± 0.13 m *vs* 1.56 ± 0.11 m; Mann–Whitney *W* = 20,369, *p* < 0.01). As with body weight, there was no relationship between body length and mean maximum dive depth for either of the sexes in the tagged animals (*r*^2^ < 0.01, *p* = 0.9; Length effect *F*_1,29_ = 0.03, *p* = 0.8; Sex effect *F*_1,28_ = 2.5, *p* = 0.1) (Fig. 5A). However, a positive relationship was observed between body length and mean dive duration for both sexes (*y* = 1.3*x* − 6.1, *r*^2^ = 0.3, *p* = 0.005; Length effect *F*_1,29_ = 9.3, *p* = 0.005; Sex effect *F*_1,28_ = 3.9, *p* = 0.06) (Fig. 5B). A strong positive relationship between mean bottom duration and body length was only found for the males (*y* = 1.5*x* − 93.4, *r*^2^ = 0.5, *p* < 0.001; Length effect *F*_1,29_ = 13.2, *p* = 0.001; Sex effect *F*_1,28_ = 5.4, *p* = 0.03) (Fig. 5C). Neither sex showed a relationship between body length and the rate of change in depth during the descent phase (Length effect *F*_1,28_ = 0, *p* = 0.9; Sex effect *F*_1,29_ = 4.1, *p* = 0.05), the bottom phase (Length effect *F*_1,28_ = 3.2, *p* = 0.08; Sex effect *F*_1,29_ = 8.8, *p* = 0.006) or the ascent phase (Length effect *F*_1,28_ = 0.01, *p* = 0.9; Sex effect *F*_1,29_ = 4.3, *p* = 0.05).

## Discussion

This study seemed to have a systematic bias in the age distributions of the captured animals that may serve to confound patterns. It is well documented that mature male harbour seals are somewhat longer and heavier than mature females (Lindenfors, Tullberg & Biuw, 2002) and our larger sample of captured animals showed this trend clearly, even though the animals that we selected for tagging showed no significant differences. We suggest that this result was due to the limited sample size with high variation and likely some unintended bias in selection of animals for tagging. As reported earlier, we ensured that only healthy animals were tagged following an extensive veterinary examination so the bias may have come from this too, especially if smaller males were in better condition than larger males. Regardless of how this bias arose, tagged males were likely to have been younger animals than the tagged females, and thus any differences in diving behaviour are unlikely to be driven by allometry effects (Schreer & Kovacs, 1997; Thompson et al., 1998). Non-equivalence in age groupings of the two genders makes our intersex comparison less than ideal, particularly if some of our animals were, unintentionally, sub-adults. We have no objective way of assessing this *post-hoc*. However, given the fact that all individuals within one area have the potential to compete, any differences between males and females in our findings might, nonetheless, point to mechanisms that reduce competition. In this regard, it is interesting to note that grey seals (*Halichoerus grypus*) begin to exhibit sex-related differences in diving even when young and essentially monomorphic, even though the sexes are dimorphic as adults (Breed, Bowen & Leonard, 2011, cf. Breed, Bowen & Leonard, 2013).

Our hypothesis, that there would be no intersex differences in depth use in harbour seals, was not borne out by the data because, despite there being no significant differences between the tagged sexes in either body length or mass, males dived deeper than females (Fig. 2A). The generally accepted phenomenon that larger species can dive deeper (Schreer & Kovacs, 1997; Thompson et al., 1998) stems from the observation that larger species can also stay submerged for longer (Schreer & Kovacs, 1997; Irvine et al., 2000), and thus have more time to reach greater depths (Halsey, Butler & Blackburn, 2006). These depth- and duration-linked allometric trends have been explained by decreasing mass-specific metabolic rate with increasing mass (Butler & Jones, 1982; Halsey, Butler & Blackburn, 2006) coupled with linearly increasing body oxygen stores with mass (Brischoux et al., 2008). In short, while the mass-specific amount of oxygen available for dives remains the same, smaller species use it faster and so cannot remain underwater as long (Halsey, Butler & Blackburn, 2006). But here, too, our results seem at odds with convention because there were significant differences in dive durations between male and female harbour seals, with females staying longer underwater (Fig. 2B), despite the fact that they reached shallower depths (Fig. 2A). Interestingly, all these patterns were apparent even though both male and female animals adhered to conventional allometric theory (see above) by generally diving longer if they were more massive (Figs. 4 and 5).

All other things being equal, the explanation for the intersex differences could, however, stem from a differential rate of energy expenditure underwater linked to differing activities so that oxygen stores were used at differential rates. In fact, the rate of change of depth data indicate a mechanism by which this mechanism could have occurred. Although absolute swim speed was not measured, the vertical velocities during the descent and ascent phases of the dive (which were derived from adjacent depth readings divided by the sampling interval) were significantly higher, by some 34%, in male than in female seals. If dive angles were the same, this finding implies that the males were actually swimming faster, and having to use more power to do so.

In order to speculate on the potential effect of this explanation, we assume, in a first iteration, that both sexes have an approximate ‘normal swim speed’ of about 1.4 m/s (Williams & Kooyman, 1985). By then using an approximate gender-common descent rate of 0.585 m/s (the mean of all values across sites and sexes), we can derive a descent angle of approximately 24.7°. These values allow us to compute the proper sex-specific descent rates to re-assess putative sex-specific swim speeds as 1.60 and 1.20 m/s for males and females, respectively (noting that this process simply translates a difference in vertical descent rate of 34% into an absolute swim speed difference of 34%). Williams & Kooyman (1985) calculated that the drag force of harbour seals was related to swim speed via the following equation:

(1) \begin{eqnarray*} \mathrm{Drag}=6.49{\mathrm{Speed}}^{1.79} \end{eqnarray*}

where drag is given in Newtons and speed in metres per second. Thus, males and females would experience drags of 15.05 and 8.99 N, respectively, at their calculated swim speeds. Gas respirometric studies examining the relationship between swim speed and power in harbour seals, give this as:

(2) \begin{eqnarray*} \text{Rate of oxygen consumption }=4.6+3.1{\mathrm{Speed}}^{1.42} \end{eqnarray*}

where the rate of oxygen consumption is given in ml/kg/min (Davis, Williams & Kooyman, 1985). Thus, these gender-specific derived speeds translate into metabolic powers of about 3.56 and 2.89 W/kg for males and females, respectively (assuming that 1 ml oxygen equates to 20.1 J; Schmidt-Nielsen, 1997). These calculations give a difference of some 23% between the sexes, which equates well with the 36% difference in the best-fit gradients in the relationship between dive duration and dive depth for the two sexes (Fig. 3: see results or regression equations). If our calculations are approximately correct, they are unsurprising because maximum dive depth modulates energy expenditure by integrating both time (and therefore energy) for the commuting period between the water surface and the preferred foraging depth as well as increasing bottom time (and therefore energy) with increasing depth. Critically, increases in both commuting (vertical) distance during the transition phases between surface and foraging depth and bottom distance with increasing depth (see Mori, 2002 and references therein) will implicate higher energy expenditure at higher speeds (see above), leading to an overall reduction in dive duration, as we observed in our data.

The observations made above assume that both males and females dive at approximately the same angle, and that swimming speeds calculated for the descent are also applied during the extensive bottom phase. Variation in either of these two parameters will alter predictions accordingly. Irrespective of the extent to which swim speed genuinely varies between male and female harbour seals, it is clear that speed is an important variable needed in discussions of niche partitioning, both inter- and intra-specifically, most particularly because the power required to swim underwater increases approximately as a cube of the swim speed (e.g., Boyd et al., 1995; Culik et al., 1996; Bethge et al., 1997). Higher swim speeds may take one gender away from minimum costs of transport, reducing dive duration, as observed here. At the same time, higher speeds would tend to take the faster gender (males) rapidly into more distant regions which should help to mitigate competition. Certainly, consideration of swim speed alone may help explain why, irrespective of body mass, in some cases it is the males that dive deeper and longer (Baechler, Beck & Bowen, 2002; Staniland & Robinson, 2010), while in other cases it is the females that do this (Beck, Bowen & Iverson, 2003; Beck et al., 2003a; Beck et al., 2003b). This suggestion may also explain inter-gender differences in foraging location (e.g., Breed et al., 2006). Thus, if our assumptions regarding our harbour seal data are correct, we would predict that males from our study colonies range farther than the females and that, given the gentle increase in depth with distance from the colonies, which may help explain inter-sex differential depth use and dive durations, and ultimately be a mechanism by which this species minimizes consumptive competition.

## Conclusions

Although harbour seal populations in northern Europe are not currently so large that severe consumptive competition might be expected, the habit of forming colonies, along with historically large population as baselines, implies that selection pressure should play some role in selecting for inter-gender behavioural or morphological differences to reduce competition. Our results showing increasing dive duration with dive depth are in line with many other pinniped studies but our observation that females have higher depth-specific dive durations than males requires an explanation. Although there were differences between males and females in our untagged population, our animals sampled for tagging had no gender-linked differences in body mass or length, both factors which are often linked to dive capacity. Interestingly, we noted higher vertical speeds in our tagged males which, if representative of swimming speeds overall, should equate to higher metabolic rates and consequently shortened dive durations. Differential travel speeds should affect a suite of features, such as ability to detect prey and changing dive:pause ratios, which translate into differential foraging ecology. Although speculative, we suggest that this phenomenon requires further investigation since it could prove an important mechanism for minimizing competition in harbour seals and other colonial pinnipeds.

## Supplemental Information

10.7717/peerj.957/supp-1 Data S1 Supplementarydata1

10.7717/peerj.957/supp-2 Data S2 Supplementarydata2
